# Algebraic correction methods for computational assessment of clone overlaps in DNA fingerprint mapping

**DOI:** 10.1186/1471-2105-8-127

**Published:** 2007-04-18

**Authors:** Michael C Wendl

**Affiliations:** 1 Genome Sequencing Center, Washington University, St. Louis MO 63108, USA

## Abstract

**Background:**

The Sulston score is a well-established, though approximate metric for probabilistically evaluating postulated clone overlaps in DNA fingerprint mapping. It is known to systematically over-predict match probabilities by various orders of magnitude, depending upon project-specific parameters. Although the exact probability distribution is also available for the comparison problem, it is rather difficult to compute and cannot be used directly in most cases. A methodology providing both improved accuracy and computational economy is required.

**Results:**

We propose a straightforward algebraic correction procedure, which takes the Sulston score as a provisional value and applies a power-law equation to obtain an improved result. Numerical comparisons indicate dramatically increased accuracy over the range of parameters typical of traditional agarose fingerprint mapping. Issues with extrapolating the method into parameter ranges characteristic of newer capillary electrophoresis-based projects are also discussed.

**Conclusion:**

Although only marginally more expensive to compute than the raw Sulston score, the correction provides a vastly improved probabilistic description of hypothesized clone overlaps. This will clearly be important in overlap assessment and perhaps for other tasks as well, for example in using the ranking of overlap probabilities to assist in clone ordering.

## Background

Fingerprint mapping continues to play an important role in large-scale DNA sequencing efforts [1-5]. The procedure is challenging in terms of both its laboratory and computational demands. Indeed, most of the computational steps involve non-trivial algorithmic aspects. While reasonable solutions have been found for many of these, one task that remains particularly problematic is assessing postulated clone overlaps based on their fingerprint similarity.

The "overlap problem", as this is often referred to, basically involves examining all pairwise clone comparisons in order to identify overlaps. For a map consisting of *λ *clones, there are *C*_*λ*, 2 _= *λ *(*λ *- 1)/2 such comparisons. In each one, the number of matching fragment lengths between the two associated fragment lists is established. A case having *μ *> 0 matches indicates a possible overlap because the mutual length(s) may represent the same DNA. Lengths are not unique, so such matches are not conclusive indicators of overlap. Instead, the problem is largely one of probabilistic classification. One or more quantitative metrics are used to evaluate the authenticity of each such case. For example, an apparent overlap might be judged against its likelihood *α *of arising by chance. Methodologies of varying degrees of rigor have been proposed for this task [6-11]. However, the so-called Sulston score, or Sulston probability *P*_*S *_has emerged as a *de facto *standard [12], in part because of its integration in the widely-used FPC program [13,14]. A liability of a number of these methodologies, including *P*_*S*_, is they assume fragment length comparisons are independent when, in fact, they are not [10,15].

Recently, the exact distribution characterizing the overlap problem was determined [16,17]. Comparisons reveal that the assumption of independence is usually a poor one and that the Sulston score systematically over-predicts actual overlap probabilities, often by orders of magnitude. Consequently, a bias arises in projects that utilize *P*_*S *_(Table 1). One chooses the significance threshold *α *to minimize erroneous decisions according to what is presumed to be the actual probabilistic description of the problem, *P*_*E*_. The alternative result using the Sulston score is an overall increase of false-negatives (Case 1). Clones having significant overlap will still be correctly detected (Case 3). Moreover, false-positives would not be increased because *P*_*S *_errs on the conservative side with respect to non-overlapping clones (Case 6). Miscalls can obviously be expected when poor values of *α *are chosen (Cases 4 and 5). However, if *α *is set too high, there will still be circumstantial cases where the correct decision is made (Case 2). These will presumably be more than offset by a higher rate of false-positives (Case 4). In summary, *P*_*S *_is not an especially good discriminant for the overlap assessment problem.

**Table 1:** Types of decisions for the biased Sulston score

Case	Scores	Overlap	Tuning of *α*	Decision Based on *P*_*S*_
1	*P*_*E *_<*α *<*P*_*S*_	Yes	Correct	Wrong (False-Negative)
2	*P*_*E *_<*α *<*P*_*S*_	No	Too High	Circumstantially Correct
3	*P*_*E *_<*P*_*S *_<*α*	Yes	Correct	Correct
4	*P*_*E *_<*P*_*S *_<*α*	No	Too High	Wrong
5	*α *<*P*_*E *_<*P*_*S*_	Yes	Too Low	Wrong
6	*α *<*P*_*E *_<*P*_*S*_	No	Correct	Correct

The drawback of *P*_*E *_is that it is rather difficult to compute and cannot be used directly in most cases. For example, current resources are not sufficient to evaluate it for most BAC comparisons or for capillary-based fingerprinting [18]. A suitable method of approximating *P*_*E *_is therefore required. Here, we propose a straightforward correlation-based approach that derives correction factors for the Sulston score. This procedure dramatically increases accuracy without incurring much additional computational effort.

## Results

The overlap problem is formally cast in terms of two clones having *m *and *n *"bands", respectively, where *m *≥ *n*. Each band represents an individual clone fragment, with its position on a gel image providing an estimate of the fragment's length. Multiple bands of roughly the same length often appear. Finite measurement resolution ± *R *allows an image of length *L *to be subdivided into *t *= 0.5 *L/R *discrete bins. The Sulston score *P*_*S *_= *P*_*S *_(*μ*, *m*, *n*, *t*) is taken as a *provisional *estimate of the probability that at least *μ *fragment matches between the two clones arise by chance. Note here that the variables (*μ*, *m*, *n*) correspond to (M, nH, nL), respectively, in notations used by the FPC program [14]. The corresponding exact probability is *P*_*E *_= *P*_*E *_(*μ*, *m*, *n*, *t*), as given in refs. [16,17]. We formulate a corrected value, *P*_*C*_, that can be both efficiently calculated and that gives substantially better estimates of *P*_*E *_than the Sulston score, i.e. |*P*_*E *_- *P*_*C*_| ≪ |*P*_*E *_- *P*_*S*_|.

**Figure 1:**
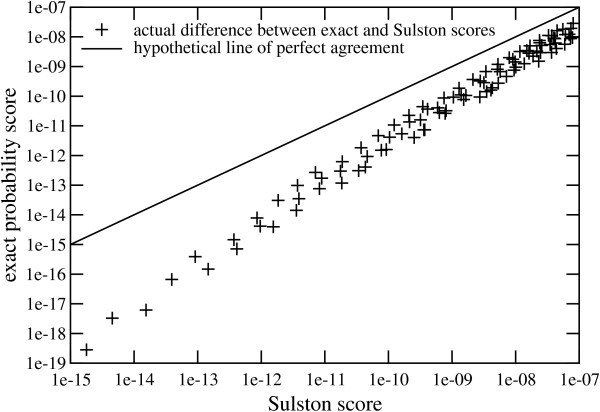
Sampling of exact probability versus Sulston score for *t *= 236.

The simple log-log plot in Fig. 1 shows good correlation (Pearson's coefficient of *ρ *≈ 0.9938), suggesting that standard regression might be a reasonable basis for correction. Note the characteristic over-prediction of *P*_*S*_. (Points representing the exact probability consistently fall below the hypothetical line of agreement between *P*_*S *_and *P*_*E*_.) These particular data are computed for *t *= 236, which describes traditional settings for fragment length measurements and comparisons, i.e. ± 7 pixels over a 3300 pixel gel image [13,19]. Considerations of coverage usually dictate a large number of clones in a map [2], so that values substantially above 10^-7 ^are not usually of interest [20]. The data range over 0 ≤ *μ *≤ *n *for a number of different fingerprint comparison sizes: 2 ≤ *n *≤ *m *for 5 ≤ *m *≤ 12, 2 ≤ *n *≤ 10 for *m *of 13 and 14, 2 ≤ *n *≤ 9 for *m *of 15 and 16, 2 ≤ *n *≤ 8 for *m *of 17 and 18, and finally 2 ≤ *n *≤ 7 for 19 ≤ *m *≤ 25. The exact solution becomes difficult to evaluate beyond these ranges using readily-available resources. Specifically, the computational effort increases according to a factor that exceeds *m*!/(*m *- *n*)! [17].

Correlation in Fig. 1 is clearly not perfect. Specifically, the points show some amount of lateral scatter. Accuracy of the correction can be further enhanced to the degree that dispersion within the window can be minimized. Here, we can apply a simple power-law data reduction model to obtain a *transformed *Sulston score

PT=PSνμξmηnζ.
 MathType@MTEF@5@5@+=feaafiart1ev1aaatCvAUfKttLearuWrP9MDH5MBPbIqV92AaeXatLxBI9gBaebbnrfifHhDYfgasaacH8akY=wiFfYdH8Gipec8Eeeu0xXdbba9frFj0=OqFfea0dXdd9vqai=hGuQ8kuc9pgc9s8qqaq=dirpe0xb9q8qiLsFr0=vr0=vr0dc8meaabaqaciaacaGaaeqabaqabeGadaaakeaacqWGqbaudaWgaaWcbaGaemivaqfabeaakiabg2da9iabdcfaqnaaDaaaleaacqWGtbWuaeaaiiGacqWF9oGBaaGccqWF8oqBdaahaaWcbeqaaiab=57a4baakiabd2gaTnaaCaaaleqabaGae83TdGgaaOGaemOBa42aaWbaaSqabeaacqWF2oGEaaGccqGGUaGlaaa@3FAF@

The four power values can be chosen empirically such that the data locally collapse into a more highly correlated set. For example, selecting (*v*, *ξ*, *η*, *ζ*) = (1.2,4,0.8,-3.4) in Eq. 1 leads to the curve-fit

PC≈9.855 PT1.171
 MathType@MTEF@5@5@+=feaafiart1ev1aaatCvAUfKttLearuWrP9MDH5MBPbIqV92AaeXatLxBI9gBaebbnrfifHhDYfgasaacH8akY=wiFfYdH8Gipec8Eeeu0xXdbba9frFj0=OqFfea0dXdd9vqai=hGuQ8kuc9pgc9s8qqaq=dirpe0xb9q8qiLsFr0=vr0=vr0dc8meaabaqaciaacaGaaeqabaqabeGadaaakeaacqWGqbaudaWgaaWcbaGaem4qameabeaakiabgIKi7kabiMda5iabc6caUiabiIda4iabiwda1iabiwda1iabbccaGiabdcfaqnaaDaaaleaacqWGubavaeaacqaIXaqmcqGGUaGlcqaIXaqmcqaI3aWncqaIXaqmaaaaaa@3D9B@

and the associated Pearson's coefficient *ρ *≈ 0.9980.

## Discussion

We propose Eqs. 1 and 2 as a correction to the standard Sulston score for typical fingerprint mapping conditions [13,19]. Although shown as two separate equations so as to clarify the concept, these can clearly be combined into a single equation for actual computations. Pearson's coefficient is not especially sensitive to the parameters in Eq. 1 and there are many combinations of (*v*, *ξ*, *η*, *ζ*) that elevate *ρ *into the ~0.998 range. Other methods for reducing the data do not perform as well as the model in Eq. 1. For example, standard dimensional analysis [21], which involves correlating variables such as *P*_*E*_/*P*_*S*_, *m*/*n*, and *μ*/*n*, cannot adequately resolve the fact that values of the individual variables relative to one another remain important.

### Accuracy assessment

Eqs. 1 and 2 are obviously straightforward to compute, leaving the question of just how much error reduction is actually realized over the un-adjusted Sulston score. This can be quantified with a simple metric. For the Sulston score, the error is taken as *E*_*S *_= |*P*_*E *_- *P*_*S*_|/*P*_*E*_. Error for the corrected result, *E*_*C*_, is calculated similarly.

**Figure 2:**
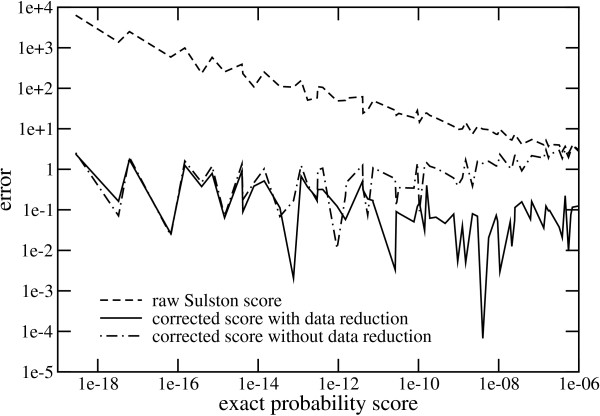
Error characterization for clones with similar numbers of fingerprint bands.

A size-selection step is part of most library-construction protocols, meaning that the variance of clone sizes will be limited to some degree. Consequently, many clone-clone comparisons will involve similar, though not necessarily equal numbers of fragments. Fig. 2 shows a comparison of error rates for the raw Sulston score and the corrected score in Eq. 2 for *m*/*n *≤ 1.3. That is, we compare clones whose numbers of fragments in their respective fingerprints are within 30% of one another. The figure also shows the error rate for the un-reduced data, i.e. for a regression equation that does not use the preliminary processing given by Eq. 1.

The Sulston score shows an increasing error as the acceptance threshold is tightened (lowered). Maximum values for the threshold are typically in the neighborhood of 10^-7 ^[20], for which *P*_*S *_over-predicts by about one order of magnitude. For threshold parameters around 10^-19^, Sulston over-prediction is about 4 orders of magnitude. While Eq. 2 shows significant local variation, the overall trend is much more constant and its error is appreciably smaller. Correction on un-reduced data also shows better accuracy than the raw Sulston score, being roughly as good as Eq. 2 up to about 10^-12^. It diverges beyond this point and eventually shows about the same level of error as the raw Sulston score. The combined correction procedure of Eqs. 1 and 2 appears to provide the best fidelity over the widest range.

### Comments on uncertainty

A simple correction of the type we propose here obviously cannot capture all the complexities inherent in the exact distribution. This results in a scatter of the data that cannot be completely eliminated, for example as illustrated in Fig. 1. This scatter is a primarily function of *m*/*n*, rather than individual values of *m *and *n*. For instance, log-log regression of data restricted exclusively to *m *= *n *returns a Pearson's coefficient of *ρ *≈ 0.9998 without *any *sort of preliminary data reduction. Of course, such a correlation would not be generally applicable to realistic clone libraries and maps.

Eq. 2 is based on the limited set of data described above. Applying it outside this set necessarily involves a degree of extrapolation, which raises two types of uncertainty. First, large *m*/*n *ratios contribute to scatter, but such extrema only emerge for cases involving sufficiently large differences between *m *and *n*. Eq. 2 accounts for data up to *m *= 25 with a maximum ratio of *m*/*n *of about 4. In the context of averages, this implies a comparison of two clones whose sizes differ by a factor of four. While there is the possibility of even greater disparities, such cases will be comparatively rare in general because of size-selection steps executed during the library-construction phase. For example, in the Human Genome Project RPCI-11 library, about two-thirds of the BAC clones were concentrated between 150 and 200 kb [22], for which the maximum *m*/*n *would be roughly 1.3. Most comparisons would be somewhat closer to one. Only about 2.5% of the library resided in each of the < 100 kb and > 250 kb ranges. This means that fewer than 0.1% of the comparisons will involve uncharacteristically large *m*/*n *ratios. Consequently, we do not view this type of uncertainty as being particularly significant.

The larger issue in our opinion arises for comparisons that extend beyond (lower than) the 10^-19 ^threshold tolerance. While minor extrapolation of a few orders of magnitude is probably not worrisome, some projects utilize substantially lower tolerances. For example, Luo *et al*. [18] and Nelson *et al*. [23] report values on the order of 10^-30 ^and 10^-45^, respectively, when using capillary electrophoresis. Other techniques, such as the traditional double-digest, can also generate higher numbers of fragments, which may require reduced thresholds. The fidelity of Eq. 2 for such cases is not clear. For example, in the data set shown in Fig. 1, larger *m*/*n *values are under-represented at the lowest scores. Because loci for larger *m*/*n *values do not slope as steeply as those for smaller ones, the trend shown in the figure may not continue in the exact same manner for values well below 10^-19^. We can only observe that the corrected score will still be the significantly more accurate choice as compared to the raw Sulston score because the assumption of independent fragment comparisons is increasingly untenable. Characterizing the exact solution in this range requires computations considerably larger than what can readily be made at present.

## Conclusion

We have calibrated Eq. 2 according to the traditional parameters used in the FPC mapping program [13]. Similar corrections can readily be constructed for different parameters. For example, protocols and software sizing methods now allow for band resolutions higher than the customary value of *t *= 236. Table 2 shows correction parameters for several such cases. Similarity of the correlation coefficients suggests that results would be comparable to that shown in Fig. 2. Although the accuracies derived from this approach are probably acceptable in the correlation range, they could, in principle, be further increased by using multiple corrections calibrated for specific "bins" of the *m*/*n *parameter.

**Table 2:** Correction parameters for various gel resolutions (bin numbers)

bins	data reduction (Eq. 1)				fit (Eq. 3)		correlation
*t*	*v*	*ξ*	*η*	*ζ*	*β*	*φ*	*ρ*

236	1.2	4	0.8	-3.4	9.855	1.171	0.9980
300	1.2	4.2	0.7	-3.2	5.070	1.144	0.9982
350	1.4	4.2	0.8	-3.2	4.908	0.956	0.9982
400	1.4	4.2	0.7	-3.2	5.711	0.944	0.9983

Clone overlap assessment is sometimes framed as a statistical testing problem [10]. Here, *α *is the probability of erroneously concluding that two clones overlap, when in fact they do not. (This casually implies that *α C*_*λ*, 2 _false positives can be expected for a project containing *λ *clones.) Consequently, corrections are most immediately relevant in the neighborhood surrounding *α *(Table 1). The overlaps here are the most valuable to detect in the sense that they are the smallest, and consequently contribute most effectively to a minimum tiling path [8]. A large fraction of the comparisons will be either far above or below the threshold, so their assessments will not ultimately be affected. However, correction is still important for these cases. For example, Branscomb *et al*. [8] have pointed out that the ability to accurately rank all overlaps according to their associated probabilities is useful in the assembly phase of mapping.

Ascertaining the degree to which a particular mapping project would actually be improved by using Sulston score correction is difficult. Aside from the usual factors that complicate comparisons [24], there are special considerations for this kind of evaluation. For example, established Sulston-based mapping projects may have obtained their best results using threshold values that would not necessarily be considered "correct" from the standpoint of the exact probability distribution (Table 1). Biologists have historically viewed selection of the Sulston threshold to be a non-trivial, library-dependent problem and often resort to empirical sampling and iteration [25,26]. Consequently, one probably cannot obtain an objective comparison by just replacing *P*_*S *_with *P*_*C *_for these cases. Another avenue, perhaps more pragmatic, would be to assess corrections on a simulated project. For example, digesting finished sequences *in silico *[27] enables one to use the resulting simulated fingerprints to see how well a map could be reconstructed. Several variations on this method are possible [28,29]. Of course, use of correction for new projects is certainly recommended.

Other issues remain unresolved. With the exception of the conditional nature of match trials, the correction in Eq. 2 is based on the same set of assumptions as the Sulston score. Neither consider, for example, possible non-IID distribution of fragment lengths or length-dependent measurement accuracy. Consequently, we feel that the simple correction procedure proposed here represents a reasonable, though admittedly provisional advance in DNA mapping methodology.

## Methods

Parameters in Eq. 1 were chosen empirically to minimize dispersion (maximize Pearson's coefficient) over a scoring range of roughly 10^-7 ^to 10^-19^. The former is often the maximum value used in a mapping project and is dictated by the need to limit false-positive overlap declarations for the associated libraries, which are typically quite large [20]. The latter is set by computational limitations.

Correction of a probability score *P*_*T *_is implemented as a so-called "power-law" algebraic expression

PC=β PTφ,
 MathType@MTEF@5@5@+=feaafiart1ev1aaatCvAUfKttLearuWrP9MDH5MBPbIqV92AaeXatLxBI9gBaebbnrfifHhDYfgasaacH8akY=wiFfYdH8Gipec8Eeeu0xXdbba9frFj0=OqFfea0dXdd9vqai=hGuQ8kuc9pgc9s8qqaq=dirpe0xb9q8qiLsFr0=vr0=vr0dc8meaabaqaciaacaGaaeqabaqabeGadaaakeaacqWGqbaudaWgaaWcbaGaem4qameabeaakiabg2da9GGaciab=j7aIjabbccaGiabdcfaqnaaDaaaleaacqWGubavaeaacqWFvpGAaaGccqGGSaalaaa@37C7@

where *φ *and *β *are regression constants. Eq. 3 can be transformed into log-log form as

ln *P*_*C *_= ln *β *+ *φ *ln *P*_*T*_.

Standard linear regression [30] can be used to determine *φ *and *β *in this equation. Specifically, we analyze the transformed system (*x*', *y*') = (ln *P*_*T*_, ln *P*_*C*_) to obtain the slope *s *and y-intercept *y*_*o *_of the straight-line equation *y*' = *sx*' + *y*_*o*_. The desired correction in Eq. 3 is then recovered by substituting *φ *= *s *and *β *= exp(*y*_*o*_).
